# Mindfulness training improves attentional task performance in incarcerated youth: a group randomized controlled intervention trial

**DOI:** 10.3389/fpsyg.2013.00792

**Published:** 2013-11-08

**Authors:** Noelle R. Leonard, Amishi P. Jha, Bethany Casarjian, Merissa Goolsarran, Cristina Garcia, Charles M. Cleland, Marya V. Gwadz, Zohar Massey

**Affiliations:** ^1^College of Nursing, New York UniversityNew York, NY, USA; ^2^Department of Psychology, University of MiamiCoral Gables, FL, USA; ^3^Lionheart FoundationBoston, MA, USA

**Keywords:** adolescent development, incarcerated adolescents, detained adolescents, stress, attention, mindfulness meditation

## Abstract

We investigated the impact of cognitive behavioral therapy and mindfulness training (CBT/MT) on attentional task performance in incarcerated adolescents. Attention is a cognitive system necessary for managing cognitive demands and regulating emotions. Yet persistent and intensive demands, such as those experienced during high-stress intervals like incarceration and the events leading to incarceration, may deplete attention resulting in cognitive failures, emotional disturbances, and impulsive behavior. We hypothesized that CBT/MT may mitigate these deleterious effects of high stress and protect against degradation in attention over the high-stress interval of incarceration. Using a quasi-experimental, group randomized controlled trial design, we randomly assigned dormitories of incarcerated youth, ages 16–18, to a CBT/MT intervention (youth *n* = 147) or an active control intervention (youth *n* = 117). Both arms received approximately 750 min of intervention in a small-group setting over a 3–5 week period. Youth in the CBT/MT arm also logged the amount of out-of-session time spent practicing MT exercises. The Attention Network Test was used to index attentional task performance at baseline and 4 months post-baseline. Overall, task performance degraded over time in all participants. The magnitude of performance degradation was significantly less in the CBT/MT vs. control arm. Further, within the CBT/MT arm, performance degraded over time in those with no outside-of-class practice time, but remained stable over time in those who practiced mindfulness exercises outside of the session meetings. Thus, these findings suggest that sufficient CBT/MT practice may protect against functional attentional impairments associated with high-stress intervals.

## Introduction

On any given day, over 100,000 youth are detained in prisons, jails, and juvenile detention centers across the United States (OJJDP, 2009). The overwhelming majority of these youth have experienced significant early psychosocial adversity which often has deleterious effects on brain development, particularly in areas of cognitive control responsible for regulating emotions (Abram et al., 2004; van Goozen et al., 2007; Ganzel et al., in press). Deficits in cognitive control processes, including attention, working memory, and regulation of emotion, are associated with behavioral disorders that are prevalent among youth offenders and contribute to the development and persistence of antisocial behavior (Teplin et al., 2002; Cauffman et al., 2005; Blair and Razza, 2007). In addition, early adversity, particularly childhood maltreatment and hostile behavior in primary caregivers, is associated with hypervigilance to hostile cues and a bias toward interpreting malevolent intent in ambiguous or neutral social situations (Dodge, 2006). Emotionally charged risk-taking situations may exacerbate these biases, particularly for youth who have challenges with cognitive control. These prevalent social and emotional characteristics in detained youth are met with a culture of bullying and violence by peers and staff in correctional facilities [Connell and Farrington, 1996; Ashkar and Kenny, 2008; New York State Juvenile Justice Advisory Group (NYSJJAG), 2010]; loneliness, boredom, and reduced autonomy (Lyon et al., 2000); and a lack of social and educational services (Ashkar and Kenny, 2008). As correctional facilities are often located far from youths' homes, detainees typically have reduced contact with family and friends (NYSJJAG, 2010). Thus, there is a need for treatment and support strategies for detained youth to improve cognitive and emotional control in the stressful detainment environment. In addition, training methods that allow youth to actively engage in exercises on their own to improve cognitive control may be ideal in conjunction with structured intervention activities or psychotherapy to help youth cultivate resilience by building their capacity for cognitive control while detained and after release.

Cognitive behavioral therapy (CBT) has garnered the greatest empirical support as a treatment modality for youth involved in the criminal justice system, although the effects of these interventions tend to be small to moderate in magnitude (Lipsey and Wilson, 1998; McCart et al., 2006; Lipsey, 2009). In the present study, we investigate if mindfulness training (MT) increases the potency of a CBT intervention that is directly targeted to youthful offenders. MT has been previously found to have salutary effects on cognitive and emotional functioning among community and clinical samples of adults and youth (Shapiro et al., 2006; Biegel et al., 2009; Segal et al., 2012) as well as high stress adult cohorts such as pre-deployment military service members (Jha et al., 2010), although its effects on detained youth has not yet been explored. Mindfulness can be described as a particular way of paying attention, on purpose, and in the present moment (Kabat-Zinn, 1990) and is often characterized as comprising two components: self-regulation of attention and non-judgmental awareness (Bishop et al., 2004). One foundational mindfulness exercise, referred to as mindfulness of breathing, involves directing attention to localized sensations related to breathing; when attention wanders, it is to be gently shifted back to the breath. Paired with instructions to focus attention, participants are guided to remain receptive and to monitor the occurrence of wandering thoughts, feelings, and sensations during the exercise by acknowledging their presence without judgment or elaboration. MT has been embedded in interventions that focus on stress reduction, such as Mindfulness-Based Stress Reduction (MBSR; Kabat-Zinn, 1990), and those that utilize cognitive behavioral treatment strategies, such as Mindfulness-Based Cognitive Therapy (MBCT; Segal et al., 2002). In MBCT, consistent training in shifting attention away from ruminative thoughts and back to the breath is postulated to break the self-perpetuating cycle of negative thinking and the downward spiral of negative mood (Segal et al., 2002). Mindfulness-based interventions have been found to improve attention regulation and adaptive coping in community samples of adults (Shapiro et al., 2006; Jha et al., 2007; Tang et al., 2007; van den Hurk et al., 2010) and, more recently, a growing body of research has examined the effects of interventions that incorporate MT for youth who have challenges regulating their emotions and behavior (Semple et al., 2005; Bögels et al., 2008; Biegel et al., 2009; Haydicky et al., 2012).

Attention is a key aspect of cognitive functioning and underlies behavior regulation (Posner and Petersen, 1990). For detained youth, improving attentional control would be highly beneficial; it is a means of increasing awareness of thoughts, feelings, and situations that may lead to offending behavior and directing attention toward interpreting environmental cues more accurately or more consistent with pro-social norms. Attention is a cognitive system that is supported by three discrete subsystems: alerting, orienting, and conflict monitoring (Posner and Petersen, 1990). The alerting network is hypothesized to acquire and maintain an alert state of preparedness and is fully mature by the age of 4 years (Rueda et al., 2004). The orienting network selects information that is most relevant for the current task and may be fully developed by age 9 or 10 (Huang-Pollock et al., 2007). The conflict monitoring network resolves the conflict between goals and performance and prioritizes among competing stimuli (Fan et al., 2002). In typically developing youth, it does not reach full maturity until early adulthood (Rueda et al., 2005; Diamond, 2006). Mindfulness training has been found to improve performance in orienting and conflict monitoring in adults (Brefczynski-Lewis et al., 2007; Chan and Woollacott, 2007; Jha et al., 2007; Slagter et al., 2007; Tang et al., 2007; Heeren et al., 2009) and in adolescents (Baijal et al., 2011). While short periods of MT have been found to improve attention regulation and cognitive control among novice practitioners (Tang et al., 2007; Jha et al., 2010), several studies with adults have observed a dose-response relationship with more practice leading to greater improvement in these domains (Carmody and Baer, 2008; Jha et al., 2010). For example, Jha et al. (2010) examined the protective effect of MT on adults' cognitive functioning during a period of stress. Pre-deployment U.S. Marine reservists participated in either an 8-week MT course or were placed in a no-intervention control group. Performance on a working memory task degraded over time in both groups. However, Marines who reported a spending more time engaging in mindfulness exercises demonstrated greater working memory capacity (WMC, akin to greater cognitive control) relative to those who did not practice or practiced very little.

Cognitive control mechanisms involving engagement of attention and working memory have been proposed to be critical for successful emotion regulation (Gross, 1998, 2002), which includes a sequence of automatic and controlled mental processes that occur prior to the height of emotional, physiological, and behavioral responses in high-emotion situations. In line with the centrality of attentional control in emotion regulation, Jha et al. (2010) found that greater improvements in WMC with MT led to greater reduction in negative mood. Thus, the promise of MT emerging from a growing literature in adults (see Lutz et al., 2009) is that it is a form of mental exercise that improves attention and working memory, which may in turn bolster regulation of cognitive and emotional processes. Yet, it is an open question of whether MT might similarly benefit attentional control in highly stressed youth.

In the current study, we examine whether a group-based, multisession cognitive behavioral therapy and MT (CBT/MT) resulted in improved attentional capabilities among adolescents incarcerated in a high-stress, high-security correctional facility in comparison to an active control intervention. Four main questions were examined: (1) Does performance degrade over time among detained youth, as might be expected given the stressful context? (2) If so, does CBT/MT protect against performance degradation? (3) If MT is helpful, is participation in intervention sessions alone sufficient or are there added benefits to engaging in MT practice outside of sessions? (4) Does being released from incarceration impact the magnitude of potential benefits in attentional performance that may come from CBT/MT?

## Methods

### Participants

We recruited 267 incarcerated male youth (age *M* = 17.4 years; *SD* = 0.71, range 16–18) from two buildings within a large urban correctional complex that houses mainly adults. Within each building, youth dormitories were assigned at random to receive either a CBT/MT intervention or an active control intervention. Only a subset of participants completed both pre- and post-training assessments (*n* = 201) due to the following reasons. As per the study protocol, participants (*n* = 24) who were transferred or released after the T1 assessment but before intervention activities began were not contacted for follow-up assessment. Participants (*n* = 28) who were later transferred to a facility where study activities were prohibited by correction officials could not be contacted for a follow-up assessment. In addition, 4 participants refused to complete the T2 assessment, 9 computer files were corrupted, and 1 participant was deported out of the country. Finally, an additional 10 participants were missing release dates, thus, a subset (*n* = 191) were included in the analyses. Between Time 1 (T1; pre-intervention) and Time 2 (T2; 4 months post-baseline) testing sessions, 123 participants were not released and 68 participants were released. The mean length of stay was 106 days (Median = 73 days, IQR = 111).

The majority of the participants (98%) were Black or Latino, reflecting the racial/ethnic composition of all youth in the facility. Self-report of offending behavior indicated that 74% of participants reported ever engaging in non-violent offenses (e.g., theft, selling drugs) and over half (54%) of participants reported engaging in violent offenses (e.g., murder, assault). Official reports of offending behavior were not available from the correctional facility for the majority of youth.

### Power source intervention

Power Source (PS) is a group-based cognitive-behavioral/mindfulness meditation intervention for youth involved in the criminal justice system (Casarjian and Casarjian, 2003). The intervention's overarching theoretical frame, the process model of emotional regulation (Gross, 1998), identifies five points at which emotions can be regulated in the emotion-generative process. These points include situation selection, situation modification, attention deployment and appraisal, cognitive change, and response modulation. PS specifically targets all five points by blending the social-cognitive change components of CBT with the attentional and response modification elements of mindfulness meditation. In PS, youth are trained to select peers and situations that decrease the likelihood of offending behavior and build skills to modify situations that might precipitate risk-taking behavior. Attentional training involves learning to appraise high-risk situations, identify environmental, social, emotional, and physiological triggers for risk-taking behaviors, and direct attention toward elements of situations that are incongruent with offending behavior. In addition, youth are trained to reappraise the meaning of situations to alter the emotional impact. Cognitive change is achieved in part via mindfulness, which is a key technique in this emotion regulation process as it trains youth to control their focus of attention toward more neutral stimuli and helps youth reappraise the meaning of a situation in a way that alters its emotional impact. Moreover, MT assists in modulating physiological responses to emotionally charged situations and choosing behavioral responses that are more socially appropriate and adaptive. Importantly, CBT and mindfulness have been described as complementary and synergistic processes where training in mindfulness may foster openness to different perspectives and set the stage for the adoption of CBT skills (Teasdale et al., 2003).

Both formal meditation practice and cognitive behavioral exercises were included in the PS group intervention. The intervention is manual-based and includes videos for demonstrating specific skills including directed meditations. Meditation exercises include sitting meditation, body scans, and walking meditations. After each meeting, youth are given reading assignments from a companion book that reiterates concepts from the intervention in the form of role model stories. Participants are also strongly encouraged to meditate outside of the group sessions and record the amount of time spent in meditation practice outside of sessions.

Youth in the control group received an evidence-based cognitive-perception intervention focusing on attitudes and beliefs about substance use and violence which was modified to exclude any skills or concepts that were under investigation in the PS intervention. Thus, the intervention controlled for time, attention, and the effects of common therapeutic factors, such as therapeutic alliance, empathic counselors, and group cohesion (Safer and Hugo, 2006; Del Boca and Darkes, 2007).

The CBT/MT and control groups met separately for approximately 750 min over the course of 3 to 5 weeks, depending on the safety and security demands of the housing areas. Each session lasted approximately 75 min and each group typically contained between 8 and 12 participants. The groups were led by two trained clinicians who received weekly clinical supervision on implementation and fidelity to the respective manuals. Youth received $5.00 in their commissary account for each group session attended. Make-up sessions were offered individually or in small groups for youth who missed sessions due to court appearances or confinement out of the dormitory for disciplinary purposes. The CBT/MT clinicians had training in mindfulness meditation and their own meditation practices. Clinicians completed quality assurance forms after every session and received weekly supervision that included a review of audiotapes of sessions. Approximately 10% of session recordings were subject to quality assurance ratings for fidelity to both the control and PS intervention and fidelity was high across both conditions.

### Procedures

Between August 2009 and April 2011, we recruited 267 incarcerated male youth from a large, urban correctional complex populated primarily by adult prisoners. Two buildings in the complex that contained dormitories for youth, ages 16–18, were used for the current study. The first building (2 dormitories) housed sentenced youth who were serving short-term sentences (up to 12 months) while the second building (5 dormitories) housed youth who were awaiting trial or sentencing. Within each building youth dormitories were assigned at random to receive either the Power Source cognitive behavior/mindfulness training (CBT/MT) intervention (youth *n* = 147 in 4 dormitories) or an active control intervention (youth *n* = 117 in 3 dormitories). The four CBT/MT dormitories contained 20, 24, 33, and 70 participants, respectively. The three active control intervention dormitories contained 17, 26, and 74 participants, respectively.

#### Recruitment procedures

Youth were approached by research staff in the common room of each dormitory for participation in the study. Youth who had at least six weeks remaining on their sentence or estimated length of stay and could complete an interview in English were invited to participate. Youth who were 18 years old, or considered emancipated if 16 or 17, signed informed consent. Youth less than 18 years of age signed informed assent, and parental consent was obtained for participation. All procedures were approved by the New York University Institutional Review Board and the New York City Department of Corrections.

#### Assessments

At T1, as part of a longer computer-based interview using audio-computer assisted self-interview format (A-CASI), participants reported demographic and background including age, race/ethnicity, and offense history. The Self Report of Offending (SRO; Huizinga et al., 1991) was used to measure offending history. Youth reported (yes/no) if they engaged one or more of 10 illegal/antisocial behaviors over their lifetime which included violent (e.g., assault, homicide) and non-violent crimes (theft, selling illegal drugs). T2 interviews occurred approximately 21 weeks (range 11–79 weeks) after the T1 interview. At T2, participants in the PS intervention used a 5-point Likert scale (“never” to “several times a day”) to report the amount of time they typically meditated outside of the intervention sessions. Participants received $25.00 in their commissary accounts (or in cash if released at T2) for participation in each interview.

### Stimuli and design

Participants completed the computerized Attention Network Test (ANT; Fan et al., 2002) at T1 and T2 which tests the efficiency of the attentional networks. In the ANT, participants are instructed to focus on a fixation cross in the center of the computer screen. At the start of each trial, a warning cue (asterisks) provides spatial and temporal information about the upcoming target. Participants are instructed to press the right or left arrow key when the target appears as quickly and as accurately as possible. There are four cue conditions. In the no-cue condition, the fixation cross remains on the screen, and the target can appear either above or below the cross; in the double-cue condition, cues appear above and below the fixation cross, and the target can appear either above or below the cross. In the center-cue condition, the fixation cross is replaced with a cue, and the target can appear either above or below the cross. In the spatial cue condition, one cue appears at the location of the target; the spatial cue was 100% predictive of the target position and was equally likely to occur above or below the fixation point. Targets were groups of five arrows pointing in the same direction (congruent), the central arrow pointing in the opposite direction (incongruent), or the solitary central arrow (neutral). The participant's task was to indicate the direction of the central arrow by responding with a left- or right-click on a mouse using the left or right index finger. After an initial practice session, all participants performed a total of 312 experimental trials which lasted approximately 8 min.

## Results

All analyses were performed on response times (RT) for correct trials and accuracy (% correct) scores. In addition to overall task performance, the efficiency of each attentional network (alerting, orienting, and conflict monitoring) was examined separately via paired RT subtractions across subsets of conditions. These analyses considered only trials on which the target was flanked by arrows, excluding the neutral target condition. Alerting was indexed by the difference between RTs on double-cue trials and no-cue trials (collapsed across target congruency condition). Orienting was indexed by the difference between RTs on spatial-cue trials and center-cue trials (collapsed across target congruency). Conflict monitoring was indexed by the difference between RTs on congruent target trials and those on incongruent target trials (collapsed across cue-type). The results of these paired subtractions will be referred to as subsystem scores. This method of analysis has been used extensively with the ANT and has been reported in detail elsewhere (e.g., Fan et al., 2002; Jha et al., 2007). We also investigated response variability by calculating the ICV (intra-individual coefficient of variation) which has been shown to decrease after intensive mediation training (Lutz et al., 2009). This coefficient provides a measure of response time dispersion relative to the mean (e.g., Reed et al., 2002; Volkow et al., 2002; Stuss et al., 2003; Bellgrove et al., 2004; Kelley, 2007). It is computed by dividing the standard deviation of response time by the mean response time for each participant. Many recent reports suggest that such response variability changes may reflect distinct processes from overall performance changes. They may be more aligned with a subjective sense of concentration vs. changes in effort, motivation, or mastery over performance (see Zanesco et al., 2013).

There were four stages of analysis. For the first stage, T1 data were examined (1) to confirm basic task effects and compare effects to prior ANT findings in this age-range (see Baijal et al., 2011) and (2) to determine whether there were baseline group differences (CBT/MT vs. control). For the second stage, analyses of variance (ANOVAs) were completed on subsystem scores and overall performance (% correct, RT) as a function of Time and Group (CBT/MT vs. control). For the third stage, the CBT/MT group was divided into those who reported practicing MT outside of intervention sessions and those who did not practice outside of intervention sessions, and ANOVAs were performed to determine if the Control, No-Practice, and Practice groups differed from each other at T2. In the last stage, we examined differences based on possible effects of environmental conditions.

### Stage 1: basic effects at T1 (pre-intervention)

Overall task accuracy was high (93%, SD = 11).

#### Subsystem performance by group

Independent *t*-tests were conducted to examine if there were group differences (CBT/MT vs. Control) at T1 on each system score (Alerting, Orienting, Conflict Monitoring).

#### Alerting

**Figure 1:**
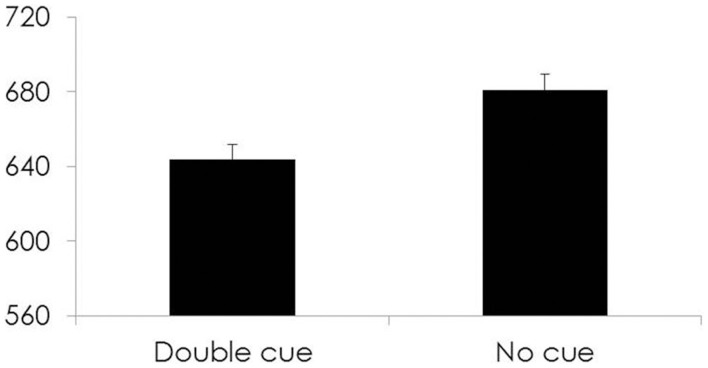
**For all participants, Time 1 Response times (ms) on the y-axis and cue type for each condition of the Alerting subsystem on the x-axis**.

The contrast between the CBT/MT group and the control group revealed no significant difference between groups in either RT [*t*_(197.58)_ = −0.57, *p* = 0.57] or % correct [*t*_(199)_ = −0.19, *p* = 0.85] scores. See Figure 1.

#### Orienting

**Figure 2:**
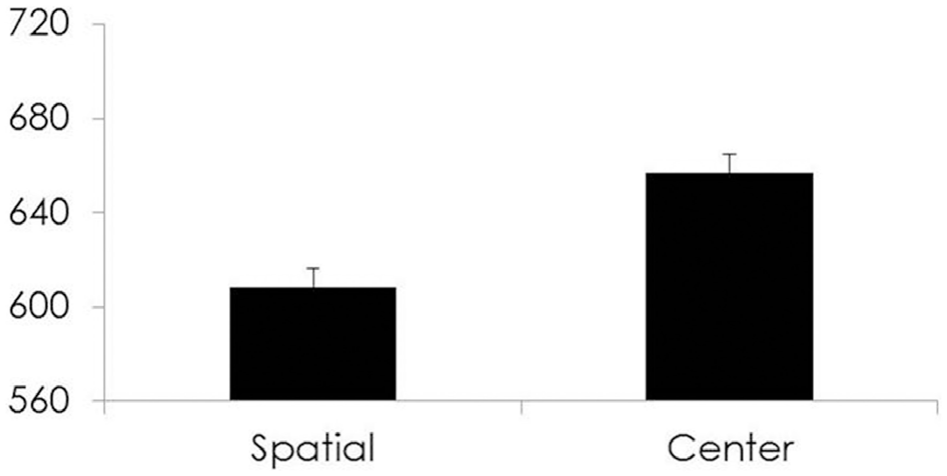
**For all participants, Time 1 Response times (ms) on the y-axis and cue type for each condition of the Orienting subsystem on the x-axis**.

There was a marginally significant difference between CBT/MT and control groups in RT difference [*t*_(199)_ = 1.68, *p* = 0.09, Cohen's *d* = 0.24] and no significant differences in % correct difference scores [*t*_(199)_ = −1.06, *p* = 0.29]. See Figure 2.

#### Conflict monitoring

**Figure 3:**
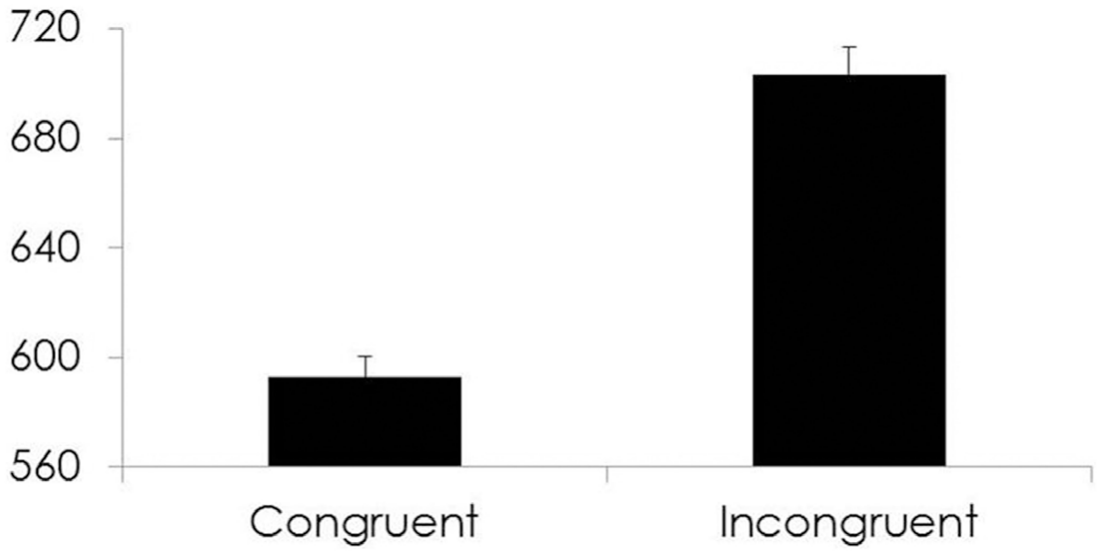
**For all participants, Time 1 Response times (ms) on the y-axis and cue type for each condition of the Conflict Monitoring subsystem on the x-axis**.

The CBT/MT group and the control group did not differ in either RT difference [*t*_(165.09)_ = −1.65, *p* = 0.10] or % correct difference [*t*_(196.34)_ = 1.32, *p* = 0.19] scores. See Figure 3.

#### Overall task performance

In addition to the system scores, experimental group and release status differences were examined via bivariate analyses. Treatment and control groups did not differ in overall % correct [*F*_(1, 199)_ = 0.81, *p* = 0.37] or RT [*F*_(1, 199)_ = 2.26, *p* = 0.13].

Thus, the magnitude of the basic effects observed for all three system scores and overall performance are in line with previous results in this age-range (Baijal et al., 2011). In addition, there do not appear to be any group-wise differences in intervention arm scores prior to the onset of the intervention.

### Stage 2: intervention-related changes over time

#### Subsystem performance by group

A series of repeated measures ANOVAs were conducted to examine the effects of intervention arm (CBT/MT vs. Control) and time (T1 vs. T2) on each system score (Alerting, Orienting, Conflict Monitoring).

#### Alerting

For Alerting, analyses of RT and % correct revealed no main effect of time [*F*_(1, 199)_ = 2.71, *p* = 0.10; *F*_(1, 199)_ = 0.06, *p* = 0.80], no main effect of group [*F*_(1, 199)_ = 0.003, *p* = 0.96; *F*_(1, 199)_ = 0.11, *p* = 0.75], and no interaction of time by group [*F*_(1, 199)_ = 0.41, *p* = 0.52; *F*_(1, 199)_ = 0.01, *p* = 0.92].

#### Orienting

For Orienting, analyses of RT and % correct revealed a main effect of time for RT only [*F*_(1, 199)_ = 7.22, *p* = 0.01, partial η^2^ = 0.04; *F*_(1, 199)_ = 0.36, *p* = 0.55], no main effect of group [*F*_(1, 199)_ = 0.46, *p* = 0.50; *F*_(1, 199)_ = 0.78, *p* = 0.38], and no interactions of time and group [*F*_(1, 199)_ = 1.18, *p* = 0.28; *F*_(1, 199)_ = 0.10, *p* = 0.75].

#### Conflict monitoring

Finally, for RT and % correct analyses of Conflict Monitoring, there was a main effect of time for RT only [*F*_(1, 199)_ = 12.73, *p* < 0.01, partial η^2^ = 0.06; *F*_(1, 199)_ = 0.66, *p* = 0.42], a main effect of group for RT only [*F*_(1, 199)_ = 3.97, *p* = 0.05, partial η^2^ = 0.02; *F*_(1, 199)_ = 1.11, *p* = 0.29], and no interaction of time and group [*F*_(1, 199)_ = 0.01, *p* = 0.93; *F*_(1, 199)_ = 0.63, *p* = 0.43]. At post-intervention testing, there were no differences between the CBT/MT and Control groups on any of the attentional subsystem scores.

#### Overall performance by group

**Figure 4:**
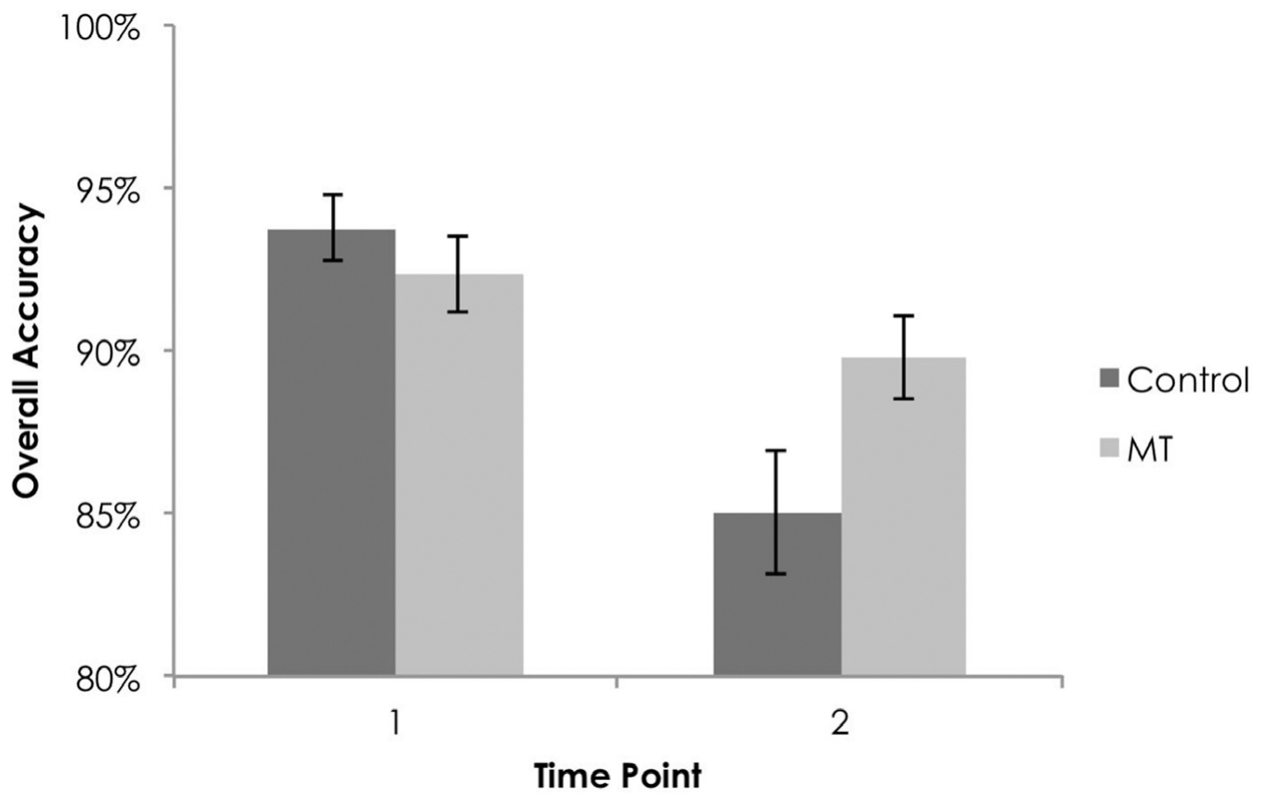
**Overall accuracy (% correct) on the y-axis and time on the x-axis as a function of training group**.

To determine if overall % correct and RT were influenced by training group over time, repeated measures ANOVAs were conducted. Examination of % correct scores revealed a significant main effect of time [*F*_(1, 199)_ = 39.16, *p* < 0.01, partial η^2^ = 0.16], no main effect of group [*F*_(1, 199)_ = 0.92, *p* < 0.34], and a significant interaction of group by time [*F*_(1, 199)_ = 11.60, *p* < 0.01, partial η^2^ = 0.06]. At T2, overall % correct was lower for both groups but higher for the CBT/MT than control group [*t*_(156.07)_ = −2.04, *p* = 0.04, Cohen's *d* = 0.30]. Analyses of RT revealed a significant main effect of time [*F*_(1, 199)_ = 11.08, *p* < 0.01, partial η^2^ = 0.05] but no effect of group [*F*_(1, 199)_ = 1.54, *p* = 0.22] and no interaction of group by time [*F*_(1, 199)_ = 0.55, *p* = 0.46]. See Figure 4.

#### Response variability by group

To examine response variability, we conducted a repeated measures ANOVA with factors of time and intervention group. Results revealed a main effect of time [*F*_(1, 199)_ = 56.75, *p* < 0.01, partial η^2^ = 0.22], no main effect of group [*F*_(1, 199)_ = 2.75, *p* = 0.10], and an interaction of time and group [*F*_(1, 199)_ = 5.75, *p* = 0.02, partial η^2^ = 0.03]. At T2, ICV was lower (responses were more stable) in the CBT/MT group compared to the control group, [*t*_(199)_ = 2.42, *p* = 0.02, Cohen's *d* = 0.34]. See Table 1 for descriptives of overall % correct, overall RT, and ICV.

**Table 1:** **Intervention-related changes over time**.

	**Control**		**CBT/MT**		**Mixed ANOVA Interaction**
	**T1 *M*(*SE*)**	**T1 *M*(*SE*)**	**T1 *M*(*SE*)**	**T1 *M*(*SE*)**	***p*-value**
Overall ACC	93.77% (0.01)	85.07% (0.02)	92.34% (0.01)	89.77% (0.01)	0.001
Overall RT	632.02 (11.45)	663.30 (12.99)	655.08 (10.17)	675.00 (10.60)	0.46
ICV	0.26 (0.01)	0.33 (0.01)	0.26 (0.01)	0.30 (0.01)	0.02

At T2, the CBT/MT group had higher accuracy and more stability in response time (ICV) than the control group.

### Stage 3: the influence of out-of-session practice on T2 performance

To examine the effect of those who engaged in out-of-session MT practice and those who did not, we looked at participants in the control arm (*n* = 87), those in the CBT/MT arm who practiced MT (*n* = 89), and those in the CBT/MT arm who did not practice (*n* = 25) in relation to changes in overall performance (% correct, RT, and ICV) given the intervention-related changes over time in these variables. Before exploring the differences between these groups at follow-up, we first checked for differences at baseline. A univariate ANOVA with baseline data from the three groups revealed significant differences in practice groups for % correct [*F*_(2, 198)_ = 2.89, *p* = 0.06, partial η^2^ = 0.03] and RT [*F*_(2, 198)_ = 3.46, *p* = 0.03, partial η^2^ = 0.03] such that the CBT/MT participants who did not practice had the lowest accuracy and the fastest RT (88%, 614 ms) when compared to the participants in the control group (94%, 632 ms) and the CBT/MT participants who practiced (94%, 666 ms).

**Figure 5:**
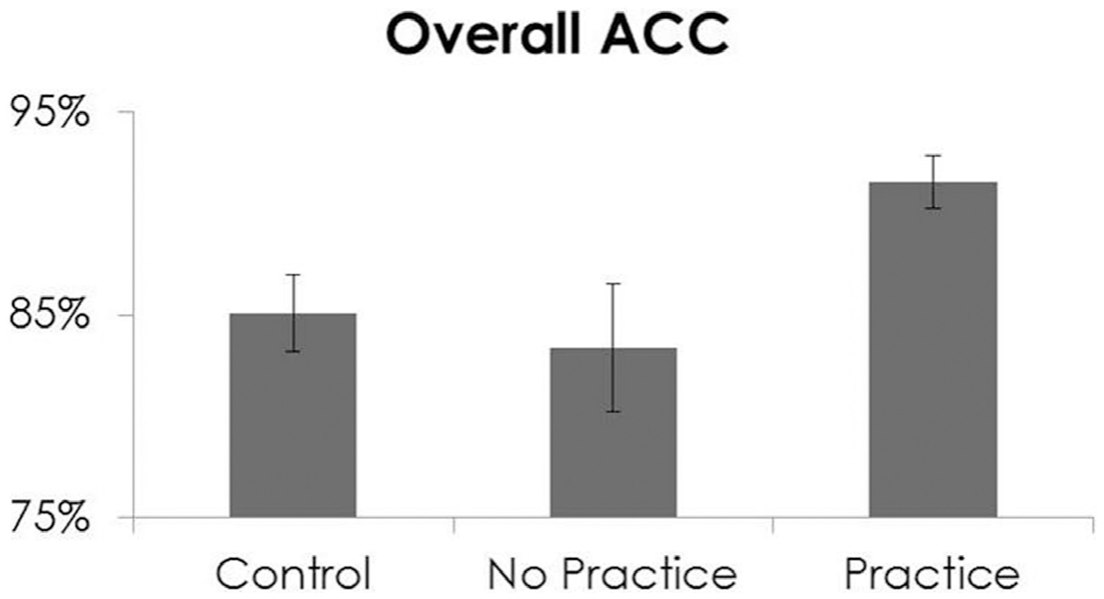
**Overall accuracy (% correct) on the y-axis and group (Controls, No Practice, and Practice) on the x-axis at Time 2 only**.

Because of these baseline differences between the practice groups, two One-Way ANCOVAs were conducted with T1 overall % correct and T1 RT as covariates. There was a main effect of practice group for % correct [*F*_(2, 197)_ = 6.59, *p* < 0.01, partial η^2^ = 0.06], such that those who practiced had better accuracy (92%) at T2 than those who did not practice (83%, effect size = 0.70) and controls (85%, effect size = 0.53). No significant effects of group were observed for RT [*F*_(2, 197)_ = 0.09, *p* = 0.92]. See Figure 5.

**Figure 6:**
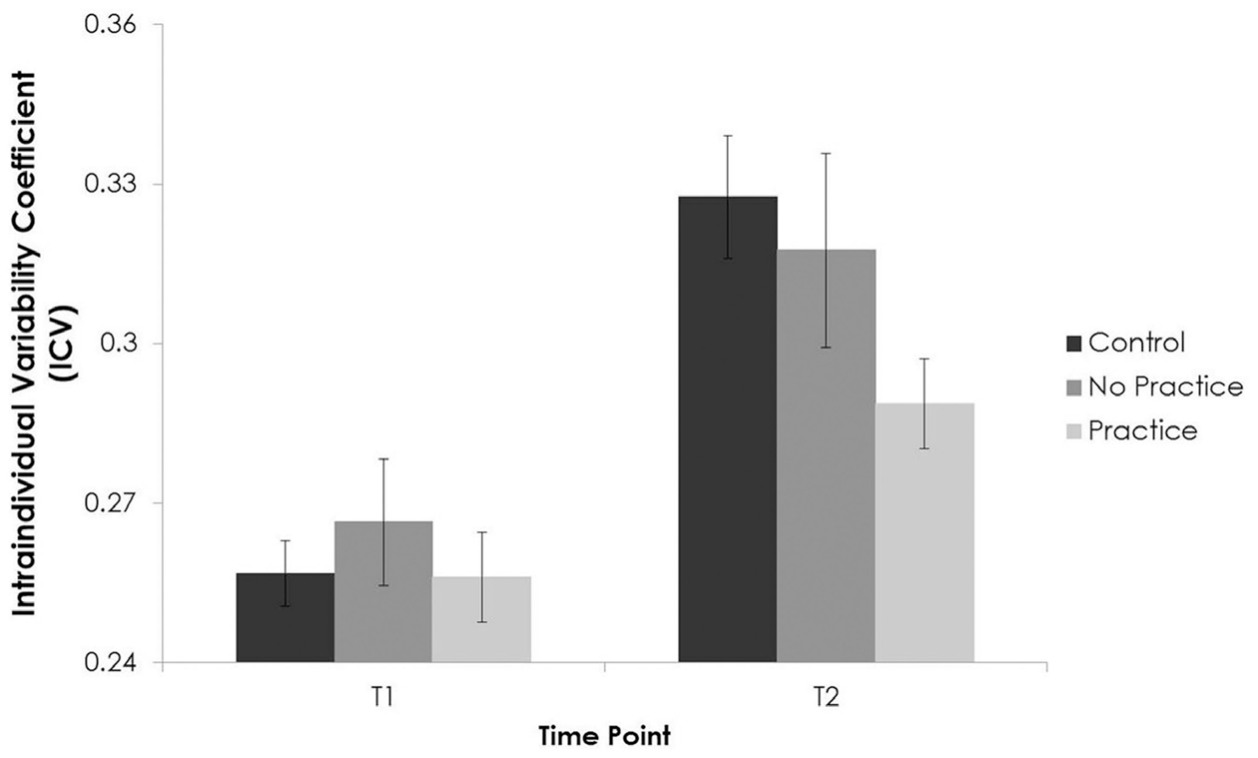
**Response variability on the y-axis and time on the y-axis as a function of group (Controls, No Practice, and Practice)**.

We also examined the effects of out-of-session practice over time on the ICV coefficient. These analyses showed a main effect of time with higher variability at T2 [*F*_(1, 198)_ = 37.84, *p* < 0.01, partial η^2^ = 0.16], no main effect of practice [*F*_(2, 198)_ = 2.26, *p* = 0.11], and an interaction of time and group [*F*_(2, 198)_ = 3.21, *p* = 0.04, partial η^2^ = 0.03]. *Post-hoc* tests (LSD) showed significant differences between control participants and participants who practiced (*p* = 0.05) with lower variability in those who practiced (0.29, effect size = 0.32) than in controls (0.33, effect size = 0.44) at T2. See Figure 6.

### Stage 4: effects of environmental conditions

#### Release status

Release information was available for only a subset of participants (*n* = 191). Before examining the possible effect of release on participants, we first compared the groups (released and detained) at baseline. Independent t-tests revealed no group differences on system scores or overall performance variables at T1, *p* > 0.05. Next, we confirmed the basic intervention-related pattern in this subset of individuals.

An ANOVA for % correct with time (T1 vs. T2) and intervention arm (CBT/MT vs. control) revealed a main effect of time [*F*_(1, 189)_ = 35.98, *p* < 0.01, partial η^2^ = 0.16], no main effect of group [*F*_(1, 189)_ = 1.49, *p* = 0.22], and an interaction of time and group [*F*_(1, 189)_ = 10.53, *p* < 0.01, partial η^2^ = 0.05]. For RT, there was a main effect of time [*F*_(1, 189)_ = 9.82, *p* < 0.01, partial η^2^ = 0.05], no main effect of group [*F*_(1, 189)_ = 2.00, *p* = 0.16], and no interaction of time and group [*F*_(1, 189)_ = 0.51, *p* = 0.48]. These results directly parallel the intervention patterns found in the group as a whole.

To determine if the release of participants from incarceration during the study period interacted with the intervention arm, an ANOVA was conducted with factors of time (T1 vs. T2), intervention arm (CBT/MT vs. control), and release group (not-released vs. released). For % correct, there was a significant effect of time [*F*_(1, 187)_ = 36.15, *p* < 0.01, partial η^2^ = 0.06], no effect of release group [*F*_(1, 187)_ = 1.61, *p* = 0.21], no effect of training group [*F*_(1, 187)_ = 1.50, *p* = 0.22], and no interaction of time, release group, and training group [*F*_(1, 187)_ = 0.77, *p* = 0.38]. For RT, there was a significant effect of time [*F*_(1, 187)_ = 11.92, *p* < 0.01], no main effect of either release status or training group [*F*_(1, 187)_ = 1.12, *p* = 0.29; *F*_(1, 187)_ = 1.46, *p* = 0.23], and no interaction of time, release group, and training group [*F*_(1, 187)_ = 0.01, *p* = 0.94].

#### Facility

Detained youth (those awaiting sentencing) and sentenced youth were held in two different buildings. To determine whether these two groups differed at baseline, we completed three univariate ANOVAs with overall % correct, overall RT, and ICV. There was no effect of facility on T1 overall % correct [*F*_(1, 199)_ = 0.39, *p* = 0.53] or T1 ICV [*F*_(1, 199)_ = 0.02, *p* = 0.90], but there was an effect of facility on T1 overall RT [*F*_(1, 199)_ = 12.09, *p* < 0.01, partial η^2^ = 0.06]. Sentenced participants were faster than those who were detained. Thus, for those measures in which T1 differences were present, ANCOVAs were conducted instead of ANOVAs to account for these baseline group differences. For those measures on which no T1 differences as a function of facility were present, ANOVAs were conducted.

Next, to examine the impact of facility over time on the control and CBT/MT arms, we conducted 2-by-2 ANOVAs for % correct and ICV and a 2-by-2 ANCOVA (with T1 RT as a covariate) for RT with facility (detained and sentenced) and group (CBT/MT and Control). For overall % correct, there was a main effect of time [*F*_(1, 197)_ = 25.47, *p* < 0.01, partial η^2^ = 0.11], no main effect of group [*F*_(1, 197)_ = 0.96, *p* = 0.33], a main effect of facility [*F*_(1, 197)_ = 3.81, *p* = 0.05, partial η^2^ = 0.02], a significant interaction of time and group [*F*_(1, 197)_ = 6.83, *p* = 0.01, partial η^2^ = 0.03], and no significant interaction of time, group, and facility [*F*_(1, 197)_ = 1.92, *p* = 0.17]. For ICV, there was a main effect of time [*F*_(1, 197)_ = 40.41, *p* < 0.01, partial η^2^ = 0.17], no main effect of group [*F*_(1, 197)_ = 2.38, *p* = 0.13], a main effect of facility [*F*_(1, 197)_ = 6.11, *p* = 0.01, partial η^2^ = 0.03], a significant interaction of time and group [*F*_(1, 197)_ = 3.93, *p* = 0.05, partial η^2^ = 0.02], and no significant interaction of time, group, and facility [*F*_(1, 197)_ = 0.02, *p* = 0.89]. Finally, for overall RT, there was no main effect of facility [*F*_(1, 196)_ = 2.25, *p* = 0.14] and no main effect of group [*F*_(1, 196)_ = 0.16, *p* = 0.69]. These patterns, namely the time by group interactions, are consistent with the intervention-related changes over time and demonstrate that facility or adjudication statuses compound the effects of the CBT/MT.

To test for possible effects due to individual dormitories, we calculated intra-class correlation coefficients (ICC) for overall performance and ANT system scores at T1 to investigate the variability within dormitories vs. between dormitories using the following formula which accounts for variable cluster sizes (Shrout and Fleiss, 1979): $\frac{MS_{\text{between}}-MS_{\text{within}}}{MS_{\text{between}}+MS_{\text{within}}\left(m_{o}\right)}$ where $m_{o}=\left[\frac{1}{k-1}\right]\left[n-\frac{\sum m_{i}^{2}}{n}\right]$, *k* is the total number of clusters, and *m*_*i*_ is the number of participants in each cluster. Because dormitory and treatment condition effects cannot be disentangled after treatment began, we only consider dorm effects at T1, before treatment.

At T1, the ICCs for % correct, RT, and ICV were 0.00, 0.09, and −0.02, respectively. The ICCs for % correct for alerting, orienting, and conflict monitoring were −0.02, 0.02, and 0.00; for RT, the ICCs were −0.04, 0.02, and 0.03. Most of these ICCs are small in size (≤0.05), with one ICC approximating a medium size ICC (0.10), as reported by (Zyzanski et al., 2004).

## Discussion

The current study is the first to demonstrate efficacy of a CBT/MT intervention for attentional functioning in high-risk incarcerated youth. Observed effects based on four lines of investigation are discussed in turn. First, we found that the high-stress period of incarceration led to degradations in attentional task performance across both groups as shown by lower overall accuracy, slower RTs, and increased response-time variability. This poorer performance over time might be accounted for by consistent stress on cognitive control, which is necessary for complex problem solving, emotion regulation, and behavioral inhibition. Unfortunately, this degradation may have negative consequences for youth as the cognitive control resources that are necessary to promote corrective behavior as resources to engage in rational (vs. reactive) decision making are increasingly unavailable. As this depletion may result in a downward spiral for high-risk youth, it is important that we investigate viable intervention strategies for this group. Since earlier research demonstrated the beneficial effects of MT on cognitive task performance in adults (e.g., Jha et al., 2007; Lutz et al., 2008) and in high-stress cohorts (e.g., Jha et al., 2010), we assessed the effects of a CBT/MT intervention for detained youth. We correctly hypothesized that degradation in attentional task performance would be attenuated in the CBT/MT group. Specifically, overall accuracy and response time variability were better in the CBT/MT group compared to the control group. These findings indicate that a CBT/MT intervention can be effective in limiting degradation in attentional performance in incarcerated youth.

Our third area of focus is based on prior studies that observed a dose-response relationship between time spent engaging in mindfulness exercises outside of the formal class context and the magnitude of performance benefits in adults. As expected, we found that those in the CBT/MT group who practiced outside of class had higher accuracy and lower response variability (greater response time stability) at T2 than those who did not practice or did not receive training. Finally, some members of the CBT/MT and control groups were released during the period of the project, but there was no significant effect of release on the pattern of results described above.

This project investigated whether an intervention program designed to bolster core cognitive control and emotion-regulation resources in individuals experiencing a protracted period of high stress might curb the degree of degradation in cognitive control. Our findings support this; however, the mechanisms of action are yet unclear. It may have been that the CBT/MT group was more motivated to engage in the task, and this basic motivation (not MT-related improvements in present-moment focus) could have produced the group-wise effects. Motivation may differ in the groups due to the content differences between the CBT/MT and control intervention. The control intervention was a cognitive-perception intervention designed to raise awareness of expectancies related to violence and substance use. In contrast, the CBT/MT intervention included emotionally-laden exercises focused on exploring familial histories of maltreatment and offending, accepting responsibility for offending behavior, and taking the perspective of the victim in their offense. Exploring these painful topics with two trusting, empathic adults who were not correctional staff may have therapeutic value that increased motivation for the task. However, we did not find differences between the CBT/MT and the control group on other tasks requiring similar levels of motivation.

Alternatively, performance in the CBT/MT group may have differed from the control group because of training in different attention skills. The CBT/MT intervention taught attention-related metacognitive skills such as reappraisal and identification of triggers for offending behavior. Training of these metacognitive skills involved repeated discussion and practice of specific ways of attending to affective, cognitive, and behavioral stimuli that place youth at risk for offending behavior. Training in mindfulness meditation involved engaging youth in discussions about their practice immediately after each sitting meditation, thereby cultivating meta-awareness of their process of attending, including when their attention wanders and their capacity to repeatedly return attention back to the breath. The intervention clinicians were experienced meditation practitioners who provided tips for maintaining engagement in meditation both in intervention sessions and outside of class. Thus, MT may have set the stage for the adoption of attention-related metacognitive skills through CBT training. Further studies should fractionate the differing possible contributions of motivation, specific intervention-related elements, and psychosocial support as well as include a direct comparison of CBT alone to the combined CBT/MT intervention with this population.

A necessary practical constraint of the study is that the group randomization was carried out at the level of the dormitories. One concern is that individuals within the same cluster might be more similar to each other than to individuals from different clusters, which could undermine standard error of the ANOVAs. To investigate such cluster variability, intraclass correlations at T1 were conducted. ICCs for most of the variables of analysis were small, with one ICC approximating a medium size for Overall RT. However, there were no significant group effects or interaction between group and time for this variable. Moreover, a small ICC is reported for variables in which we report a significant effect. Thus, it is unlikely that the pattern of findings is a result of intraclass correlations at the level of the dormitory. Therefore, the findings from the current study are well-suited to provide estimates of effect sizes and T1 ICC estimates that are essential to planning a larger cluster-randomized study.

Although our results are in line with the view that MT may be protective, there is a lack of specificity in effects on the subsystems of attention. Prior studies suggest that MT improves selective attention more so than receptive attention in novice practitioners (see Jha et al., 2007). Yet, the orienting system scores, which index input-level selection mechanisms, and the conflict monitoring scores, which reflect response-related selection mechanisms, did not vary by group or by time. Instead the benefits of CBT/MT were observed only in response variability and overall accuracy. It is important to note that the lack of MT-related change in subsystem scores cannot be explained by task insensitivity. At T1 and T2, the overall effects for each subsystem were in line with previous studies (Baijal et al., 2011). Accuracy was higher and RTs were faster for the double cue vs. no cue trials (used to index the alerting system), for the spatial cue vs. center cue (orienting system), and for the congruent vs. incongruent trials (conflict monitoring system). One point to consider is the particular direction given during particular MT exercises. Because the practice instruction for the foundational exercise of mindfulness of breathing is to pay attention to the present moment, it may be that CBT/MT participants were better able to maintain present-moment awareness *for all trials* relative to controls, and this deliberate and voluntary general attention to the task may have resulted in better performance in that group. Thus, further research is required to better understand the mechanisms by which this program was beneficial.

Nonetheless, better performance in the training group suggests that offering this CBT/MT intervention to incarcerated youth may be beneficial, and the degree of benefit does not seem to differ based on release status over the course of the program. It is notable that the degradation in performance did not remit once youth were released, highlighting the long-lasting, harmful effects of incarceration on youth. If these results are due to improvements in present-moment focus tied to the MT practices that were engaged during the intervention, the benefits of training may go far beyond our laboratory measures of attentional task performance. Many studies have reported that greater attentional capacity and WMC are tied to improved rational decision making, better emotion-regulation, behavioral inhibition, reduced impulsivity, and better psychological health (see Schmeichel et al., 2008). Future studies will be required to determine if MT-related sensitivity in laboratory-based measures correspond to real-world indices of intervention effectiveness, such as rates of recidivism and long-term psychological health.

In sum, the current study suggests that a multi-session CBT/MT intervention exerted a protective effect on offending youths' functional attentional impairments during incarceration in a high-security urban jail. Though performance on an attention control task degraded over time among youth in both the CBT/MT and control groups, the magnitude of performance degradation was significantly less in the CBT/MT condition. Moreover, within the CBT/MT group, performance degraded over time in those with no outside-of-session practice time, but remained stable over time among youth who practiced mindfulness outside of intervention sessions. Although this was a quasi-experimental study, to our knowledge, this is the first active-controlled study of the effects of CBT/MT for youth involved in the criminal justice system and it adds to the small but expanding body of research on the protective aspects of contemplative practice for youth in highly stressful situations.

### Conflict of interest statement

The authors declare that the research was conducted in the absence of any commercial or financial relationships that could be construed as a potential conflict of interest.
